# A Comprehensive Review of the Multitarget Effects of Subinhibitory Concentrations of Essential Oils Against *Salmonella*

**DOI:** 10.1111/1541-4337.70418

**Published:** 2026-02-16

**Authors:** Carolina Ramos, Yhan S. Mutz, Leticia Tessaro, Bruno Dutra da Silva, Marciane Magnani, Carlos Adam Conte‐Junior

**Affiliations:** ^1^ Analytical and Molecular Laboratorial Center (CLAn), Institute of Chemistry (IQ) Federal University of Rio de Janeiro (UFRJ) Rio de Janeiro Rio de Janeiro Brazil; ^2^ Laboratory of Advanced Analysis in Biochemistry and Molecular Biology (LAABBM), Department of Biochemistry Federal University of Rio de Janeiro (UFRJ) Rio de Janeiro Rio de Janeiro Brazil; ^3^ Center for Food Analysis (NAL), Technological Development Support Laboratory (LADETEC) Federal University of Rio de Janeiro (UFRJ) Rio de Janeiro Rio de Janeiro Brazil; ^4^ Graduate Program in Biochemistry (PPGBq), Institute of Chemistry (IQ) Federal University of Rio de Janeiro (UFRJ) Rio de Janeiro Rio de Janeiro Brazil; ^5^ Laboratory of Microbial Processes in Foods, Department of Food Engineering, Center of Technology Federal University of Paraíba João Pessoa Paraíba Brazil

**Keywords:** biofilm, efflux pump, essential oils, quorum sensing, *Salmonella*

## Abstract

Essential oils (EOs) exhibit antimicrobial activity against foodborne pathogens due to their chemical composition. This review investigates the mechanisms of action of EOs at subinhibitory concentrations against *Salmonella* strains. Even at subinhibitory concentrations, EOs induce cellular stress and modulate bacterial metabolism. EOs interfere with quorum sensing (QS) by downregulating genes (*luxS*, *pdfs*, *sdiA*, and *qseB*), which are essential for the system's function, affecting biofilm formation and virulence expression. Additionally, EOs act as antibiofilm agents, reducing cell adhesion and biomass of mature biofilms. At the molecular level, they regulate genes associated with biofilm formation (*csgA*, *csgB*, and *csgD*) and bacterial motility (*motB*, *flhD*, and *fliZ*). The combination of EOs with conventional antibiotics has shown synergistic effects. A proposed mechanism for this action is the inhibition of efflux pumps, which favors the retention of antimicrobial agents inside the cell. Despite promising results, challenges such as the sensory impact of high EOs concentrations and the need for further standardization in their application remain. Future research should focus on the long‐term effects of subinhibitory concentrations of EOs, bacterial molecular response mechanisms, and the development of new delivery systems to optimize the efficacy of EOs.

## 1 Introduction

Food contamination by *Salmonella* is a public health concern worldwide due to the pathogenicity of this microorganism and its ability to withstand adverse environmental condition (CDC 2023; Mutz et al. 2020). Currently, more than 2500 *Salmonella* serotypes are known, with *S*. Typhi, *S*. Typhimurium, and *S*. Enteritidis being the most relevant (Santos et al. 2019). Among them, *S*. Enteritidis and *S*. Typhimurium are the most frequently isolated serotypes from contaminated foods, accounting for approximately 93.8 million cases of salmonellosis annually (Majowicz et al. 2010). Clinical manifestations of the infection range from mild gastroenteritis to severe forms such as bacteremia, which can lead to death in extreme cases (Besser 2018; Lamichhane et al. 2024).

The persistence of *Salmonella* in different environments can be attributed to its ability to adhere and form biofilms on biotic and abiotic surfaces, making pathogen inactivation more challenging (Alenazy 2022; Zhang et al. 2024). The biofilm consists of bacterial cells embedded in a self‐produced extracellular matrix that offers protection against external agents. Additionally, other factors contribute to *Salmonella*’s antibiotic resistance, such as the presence of efflux pumps that expel antimicrobial compounds from the cell interior (Alenazy 2022; Zhang et al. 2024). Another critical factor is the quorum sensing (QS) system, which is a cell‐to‐cell communication system controlled by signaling molecules in response to population density, which can modulate the release of virulence factors, bioluminescence, biofilm formation, and others (Raju et al. 2022; Zhang et al. 2022a). Together, these mechanisms make *Salmonella* a highly adaptable bacteria, making it even more challenging.

In this challenging context, essential oils (EOs) have emerged as a promising alternative, presenting a broad spectrum of antimicrobial action (Bajpai et al. 2012; Hao et al. 2022). The regulatory status of EOs, generally recognized as safe (GRAS) by the FDA, is a crucial factor to facilitate their integration into food safety strategies, especially as natural alternatives to synthetic preservatives. Nevertheless, EOs applications must consider the antimicrobial efficacy, sensory impact, and safety under specific dosage use conditions. EOs have characteristic aromas and flavors, and are extracted from various plant parts such as flowers, leaves, and roots (Burt 2004). They are rich in bioactive compounds, including terpenes, phenols, and aldehydes, that can act on multiple molecular targets simultaneously, reducing the likelihood of resistant strain development (Dhifi et al. 2016; Falleh et al. 2020; Jackson‐Davis et al. 2023; Janotto et al. 2023).

However, achieving the desired antimicrobial efficacy often requires EOs to be used at concentrations higher than the minimum inhibitory concentration (MIC), which may compromise the sensory qualities of food products (Leite de Souza 2016). To balance sensory acceptability and antimicrobial efficacy, the use of subinhibitory concentrations combined with other strategies has been studied as a viable alternative (De Azeredo et al. 2011). Nevertheless, the effects and potential consequences of exposing *Salmonella* to these sub‐MIC levels have not yet been fully explored.

Given this context, it is important to understand the impacts of using EOs at subinhibitory concentrations on the physiology and behavior of *Salmonella* strains. Therefore, this review aims to comprehensively examine the antibacterial effects of EOs and their mechanisms of action at subinhibitory concentrations. In addition, it seeks to discuss the role of EOs in interfering with the QS system, preventing biofilm formation, and modulating antibiotic resistance. This review thus aims to fill this gap in the literature and provide insights that may contribute to the development of innovative approaches for controlling foodborne pathogens, with potential applications in the food industry.

## 2 EOs: An Overview of Their Mechanisms of Action

The antimicrobial activity of various EOs has been widely demonstrated against bacteria frequently associated with foodborne diseases, such as *Salmonella* spp., *Escherichia coli*, *Staphylococcus aureus*, *Listeria monocytogenes*, and *Bacillus cereus* (Coimbra et al. 2022; Kang et al. 2018; Selim et al. 2022; Sharifi et al. 2021; Zhang et al. 2016). These EOs possess broad‐spectrum antimicrobial properties, acting on multiple cellular targets. The main mechanisms of action are summarized in Table 1.

**TABLE 1:** Mechanisms of antibacterial action of EOs and their components.

Source of EO or chemical component	Mechanism of action	Description/effect	References
*Listea cubeba* Rosemary	Cell membrane disruption	Leakage of electrolytes (Na^+^, K^+^, Ca^+^), proteins, and nucleic acids → cell death	Mei et al. (2020) and Hao et al. (2022)
Thymol Carvacrol Limonene Cinnamaldehyde Eugenol	Alteration of lipid composition	Changes in fatty acid profile and increased membrane fluidity	Di Pasqua et al. (2006)
*Cinnamomum* spp.	Inhibition of ATP synthesis	Reduced ATPase activity → decreased energy production	Zhang et al. (2022b)
*Origanum vulgare* Carvacrol Thymol	Inhibition of DNA synthesis or DNA damage	Interference with DNA replication and transcription	Barbosa et al. (2020)
*L. cubeba*	Inhibition of protein synthesis	Disruption of essential cellular processes	Wang et al. (2025a)
*Lippia origanoides* *Artemisia dracunculus*	QS interference	Disruption of bacterial; communication and regulation of key genes	Guillin et al. (2021) and Pelarti et al. (2021)

**FIGURE 1:**
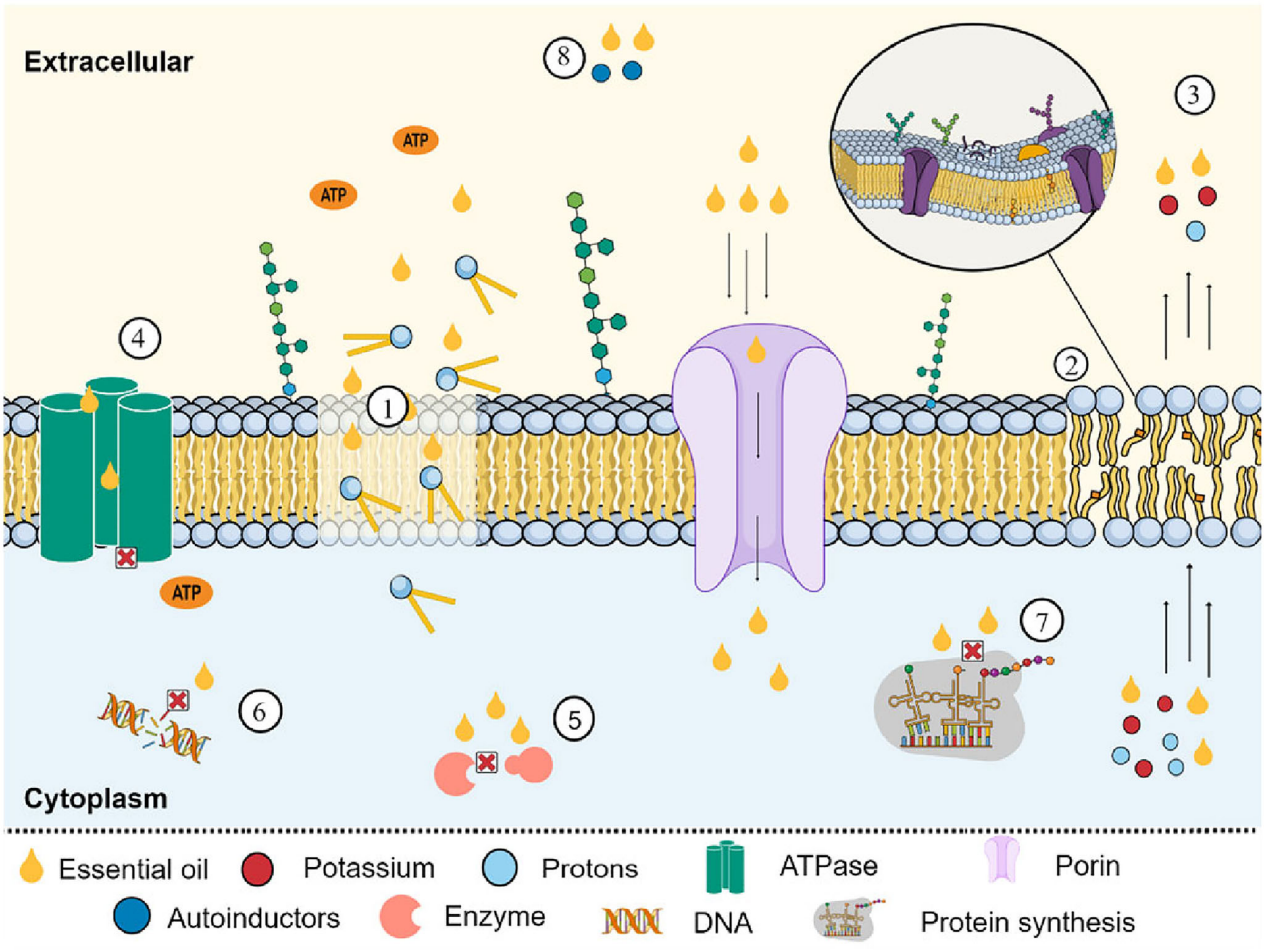
Potential mechanisms of action of EOs against bacteria. (1) Damage to the cell membrane: EOs can disrupt the lipid bilayer of the cell, affecting its integrity and causing ruptures, which compromises membrane function. (2) Modification of membrane lipid structure: Changes in fatty acid composition of the membrane, leading to alterations in fluidity and permeability. (3) Increased membrane permeability: Increased permeability allows the leakage of ions and protons, destabilizing the cell's ion homeostasis. (4) Inhibition of ATPase activity: Inhibition of ATPase reduces the cell's ability to produce ATP, impairing essential cellular functions. (5) Interference with enzymatic activity: EOs can bind to enzymes, altering their conformation and inhibiting their activity, affecting essential metabolic processes. (6) DNA damage: Some components of EOs can cause strand breaks or modify nucleotide bases, compromising replication and transcription. (7) Inhibition of protein synthesis: EOs can interfere with the ribosomal machinery, preventing the translation of proteins essential for bacterial survival. (8) Interaction with the quorum sensing (QS) signaling system: EOs can disrupt communication between bacteria, affecting their cooperative behavior, which is essential for biofilm formation and virulence factor expression.

The EOs’ main mechanism of action involves the disruption of the cell membrane structures, making them more permeable (Nazzaro et al. 2013). Due to the lipophilic nature of their constituents, EOs can cross the phospholipid bilayer, destabilizing the cytoplasmic membrane. This structural disorganization causes the leakage of essential intracellular components, including electrolytes (Na^+^, K^+^, Ca^2^^+^), nucleic acids, and proteins (da Silva et al. 2021; Figure 1).

In response to carvacrol exposure, this compound can alter membrane fluidity by modifying its fatty acid composition, contributing to the leakage of intracellular components, resulting in cell lysis and death (Di Pasqua et al. 2006). The membrane potential also plays a significant role in this process. Depolarization, caused by ion leakage, dissipates the ionic gradient and increases transmembrane ionic permeability (Benarroch and Asally 2020; Hammer and Heel 2012). Indeed, a membrane depolarization was observed in *E. coli* and *S. aureus* after treatment at the MIC with cinnamon EO (Zhang et al. 2016).

Moreover, EOs also affect ATP production (Benarroch and Asally 2020; Hammer and Heel 2012). By inhibiting ATPase activity, they reduce ATP synthesis and promote its hydrolysis, depriving the cell of the energy needed for vital metabolic processes (Zeng et al. 2021). For instance, when investigating the effect of cinnamon EO on ATP content in *S*. Enteritidis, a significant decrease was observed in the treated groups (Zhang et al. 2022b).

Intracellular effects of EOs have also been widely documented. Different concentrations of *Syzygium aromaticum* EO, equivalent to 0.25, 0.5, 1, 2, and 4 times their MIC, were shown, by macromolecular assays, to block DNA and protein synthesis in *S. aureus*. Thus suggesting that EOs may act on DNA transcription processes to mRNA and mRNA translation to protein synthesis, or even DNA replication (Xu et al. 2016). A similar effect was observed with *Origanum vulgare* EO in *S*. Enteritidis (Barbosa et al. 2020). These results indicate that EOs can cross the bacterial membrane, interact with the genetic material, and induce bacterial death.

Additionally, intracellular bacterial target involved in QS regulation are also reported as mechanisms of action for EOs (Cáceres et al. 2020; Guillín et al. 2021; Pelarti et al. 2021). These specific effects will be discussed in the following sections (Section 3.1).

## 3 Investigating the Impact of Subinhibitory Concentrations of EOs

EOs have shown potential in inactivating foodborne pathogens due to their action on multiple targets. In vitro studies suggest that this multifaceted capacity makes it difficult for bacteria to acquire resistance, even with continuous use, which may enhance their effectiveness over time (Evangelista et al. 2023). Since most EOs components are lipophilic, their interaction with bacterial cell membranes destabilizes their polarity and permeability, making it harder for resistance mechanisms to develop (Hammer and Heel 2012; Yap et al. 2014).

The antimicrobial activity of EOs is typically assessed through MIC and in vitro bactericidal assays. However, it is also crucial to investigate their effects at subinhibitory concentrations, which makes for a more realistic applicability for food and food processing. The characteristic aroma of EOs can limit their acceptance as food preservatives, as concentrations near and above the MIC may lead to undesirable flavors and odors (da Silva et al. 2022). Moreover, interactions with food constituents can also reduce their efficacy, requiring higher doses to achieve the desired antimicrobial activity (Nazzaro et al. 2013). Moreover, investigating the effects of subinhibitory concentrations is essential because bacterial cells exposed to mild damage can recover and develop changes in virulence or resistance, which could represent a problem for EOs application (Leite de Souza 2016; Silva‐Angulo et al. 2014). However, previous studies on the exposure of *S*. Typhimurium ATCC 14028 to subinhibitory concentrations of *O. vulgare* and carvacrol did not observe an increase in resistance (da Silva Luz et al. 2012). An earlier review by Leite de Souza (2016) also found no evidence of increased or decreased susceptibility to EOs or antibiotics after exposure to sublethal concentrations, attributing the absence of tolerance to the diversity of antimicrobial mechanisms in action. The consistent findings pointed to the observed changes being transient, such as alterations in membrane lipid composition (Hammer and Heel 2012). However, additional evidence suggests that this hypothesis may be valid only for short‐term exposure studies.

Despite the antimicrobial potential demonstrated by EOs, the results on bacterial resistance after exposure to subinhibitory concentrations are conflicting. Some studies, such as those by da Silva Luz et al. (2012), found no increase in resistance in foodborne pathogens after exposure to these EOs, suggesting that continuous use of these concentrations does not necessarily lead to resistance development. However, other studies, such as those by Berdejo et al. (2019) and Chueca et al. (2018), indicated that bacterial mutations may occur, leading to resistance development.

In this sense, observations on thymol reinforce this complexity, although the compound has proven highly effective in eradicating *Salmonella* biofilms on different surfaces, prior exposure to a subinhibitory concentration (78.1 ppm) resulted in a significant increase in cell resistance, characterizing a coadaptation effect. Furthermore, cells adapted to thymol also exhibited cross‐resistance to benzalkonium chloride, a widely used biocide (Strantzali et al. 2021). This phenomenon has been linked to changes in the lipid composition of the membrane and the overexpression of stress proteins ((Di Pasqua et al. 2006; Dubois‐Brissonnet et al. 2011), which can decrease cell permeability and impede antimicrobial action. These discrepancies may be attributed to factors such as experimental conditions, the bacterial strains tested, and the different mechanisms of action of the EOs, which may act differently depending on the pathogen. Therefore, the interpretation of results should consider these variables, which may explain the observed discrepancies.

Ferreira de Melo et al. (2020) investigated the exposure of *S*. Typhimurium PT4 to sublethal concentrations of *Mentha piperita* for up to 252 h. Flow cytometry analyses showed that continuous exposure to subinhibitory concentrations of *M. piperita* EOs resulted in a reduction in the number of cells with depolarized and permeabilized membranes as exposure time increased. According to the authors, this continuous exposure induced the emergence of new bacterial subpopulations with altered physiological functions that were able to preserve membrane integrity but became noncultivable, as no increase in viable counts was observed. However, mutations in bacterial populations after prolonged cyclic exposure to subinhibitory doses of EOs components led to resistant *E. coli* and *S. aureus* mutants, resulting in increased bacterial resistance (Berdejo et al. 2019; Chueca et al. 2018).

Although the literature provides a comprehensive view of the effects of EOs on food microbiota, research on their subinhibitory concentrations still lacks a deeper analysis of the potential long‐term consequences and the development of bacterial resistance. The studies found in the literature only investigated the hypothesis of acquired resistance in a short‐term framework. The resistance acquisition should be approached under the design of evolution studies, where a large time frames that encompass a large number of generations can be observed. Additionally, future research should, in these long time frames, focus on identifying the molecular mechanisms underlying these bacterial responses, considering the possibility of evolution through selective pressure and the rise of resistant subpopulations with distinct genotypic and phenotypic traits. Given the antimicrobial potential of EOs at subinhibitory concentrations and the lack of information on their effects under these conditions, investigating this aspect is crucial. These studies are essential for the safe selection of EOs as a sustainable conservation option with a perspective of whether or not they can lead to a rise of a new risk in food safety, while overcoming their sensorial and practical limitations. The following sections will further discuss the known impacts of subinhibitory concentrations of EOs on *Salmonella* cells.

### 3.1 Quorum Sensing in *Salmonella*: Mechanisms and Inhibition Strategies Using EOs

QS is the communication system among bacteria, allowing for information sharing, such as cell density, the need for specific enzymes to hydrolyze nutrients or break down toxic components, and gene expression adjustments accordingly (Yi et al. 2021). QS modulates a variety of phenotypes in bacteria, such as biofilm formation, expression of virulence factors, production of secondary metabolites, competence for DNA uptake, and bioluminescence, among other physiological processes (Whiteley et al. 2017).

The QS system operates by synthesizing and releasing signaling molecules called autoinducers. Autoinducers are released into the extracellular environment and accumulate as the bacterial population density increases (Schauder et al. 2001). When the concentration of autoinducers reaches a specific threshold, they bind to corresponding QS receptors, triggering a coordinated response that alters gene expression (Yi et al. 2021).

The QS system can be identified among Gram‐positive and Gram‐negative bacteria (Mukherjee and Bassler 2019). In *Salmonella*, it can be classified mainly into three categories based on signaling molecules: (1) Autoinducer‐1 (AI‐1), which facilitates communication among individuals of the same species in Gram‐negative bacteria, also known as N‐acyl homoserine lactones (AHLs). (2) Autoinducer‐2 (AI‐2) triggers communication between species and within the same species in Gram‐positive and Gram‐negative bacteria, commonly referring to the furanosyl borate diester. (3) Autoinducer‐3 (AI‐3), responsible for interaction with the human gut and its microbiota (Mi et al. 2024).

The AI‐1 cell density detection system comprises two main elements: the proteins LuxI and LuxR. LuxI is crucial in synthesizing the AHL autoinducer molecule (Walters and Sperandio 2006). At the same time, LuxR is activated by the extracellular presence of AHL, leading to the regulation of various genetic transcriptions and phenotypic changes. *Salmonella* does not have the LuxI homolog encoded in its genome and cannot produce AHL (Sholpan et al. 2021). Despite this, it has been observed that *Salmonella* possesses a version of LuxR, known as a Suppressor of Cell Division Inhibition A (SdiA), which allows the bacterium to detect AHLs produced by other bacterial species (Pacheco et al. 2021). The functions mediated by *Salmonella*’s SdiA are mainly related to biofilm production and virulence factors. When there is binding between SdiA and external AHLs, two genetic loci are positively activated: the *pefI‐srgC* Operon (plasmid‐encoded fimbriae–sdiA‐regulated gene) on the *rock locus* and the *srgE* gene (Mi et al. 2024).

AI‐2 refers to R‐ or S‐2‐methyl‐2,3,3,4‐tetrahydroxytetrahydrofuran (R‐ or S‐THMF). AI‐2 is produced from a precursor, 4,5‐dihydroxy‐2,3‐pentanedione (DPD), through the bacteria's LuxS/Pfs pathway (Pereira et al. 2013). The LuxS substrate is S‐ribosyl homocysteine (SRH), which LuxS uses to produce homocysteine and DPD, which can undergo spontaneous cycling to generate AI‐2 as a final product (Escobar‐Muciño et al. 2022; Mi et al. 2024). This AI‐2 system in *Salmonella* can regulate the expression of flagella‐associated genes (*fliC* and *fliD*) and SPI‐1 (*Salmonella* pathogenic island‐1) virulence factors (*invFf*, *sicA*, *sopB*, and *sopE*; Choi et al. 2012; Mi et al. 2024; Zhang et al. 2022a). Besides the intracellular production and secretion, *Salmonella* also takes up AI‐2 from the environment through the LuxS regulation system using an ATP‐binding cassette transporter (ABC‐transporter) named Lsr (Taga et al. 2001; Zhang et al. 2022a). Within the *lsr* operon, the protein LsrR is a repressor protein that can be inactivated by absorbed or synthesized AI‐2 phosphorylated by LsrK (a signaling kinase), leading to the operon transcription (Taga et al. 2001; Whiteley et al. 2017). Moreover, the LsrR protein is known to reduce the expression of SPI‐1 and genes related to flagellar motility without AI‐2 (Choi et al. 2012). Therefore, it reduces the ability of *Salmonella* to invade epithelial cells, as flagella and several precisely regulated fimbriae are essential for its motility and effective invasion (Mi et al. 2024).

The AI‐3 signaling molecule in *Salmonella* plays a vital role in regulating virulence and responding to host conditions as it captures signals produced by prokaryotic and eukaryotic cells through epinephrine (Epi) and norepinephrine (NE; Zhang et al. 2022a). AI‐3 analogs belong to a pyrazinone family of metabolites found in Gram‐positive and Gram‐negative bacteria, including *Salmonella* (Kim et al. 2020; Mi et al. 2024). AI‐3, Epi, and NE are detected by a specific histidine sensor kinase (HK) QseC that binds Ai‐3 Epi and NE, located in the cytoplasmic membrane, which is part of a two‐component system (TCS) QseBC, along with the cytosolic response regulator (RR), QseB (Ji et al. 2017; Kim et al. 2020). When HK perceives environmental signals, it undergoes autophosphorylation and transfers the phosphate group to aspartic acid residues in its free RR counterpart (Jung et al. 2012; Karavolos et al. 2008). This RR acts as a transcription factor that facilitates signal transduction by binding to DNA and modulating gene expression (Mi et al. 2024).

Another TCS system related to QS, named QseEF, has been observed in *Salmonella*. QseE acts as this system's HK, sensing Epi, NE, sulfate, and phosphate instead of AI‐3, while QseF is the RR (Kumar et al. 2020; Reading et al. 2007). Many genes are regulated by AI‐3, including T3SSs and effectors from SPI‐1 and SPI‐2 with both HKs, QseC, and QseE in *Salmonella* involved in the expression of genes associated with motility (*flhDC*), invasion capacity (*sifA*), and virulence factors (*sipA*, *sopB*, and *sifA*; Bearson and Bearson 2008; Clarke and Sperandio 2005; Rasko and Sperandio 2010).

As presented, *Salmonella* is a resourceful bacterium with intricately coordinated systems to regulate populational survival in harsh environments through QS. In the food industry, specifically, strains of *S. enterica* pose a significant risk to public health due to their high pathogenicity and survivability (Ehuwa et al. 2021). Furthermore, their ability to form biofilms on food surfaces and processing equipment further exacerbates this concern (Liu et al. 2023b). As QS controls the formation of biofilms and the regulation of virulence factors, the need for control strategies directly approaching QS inhibition is gaining much attention in the scientific community.

More specifically, the study of quorum‐sensing inhibition (QSI) has emerged as a viable therapeutic strategy in contrast to conventional antibiotics (Lu et al. 2022b). Traditional antibiotics primarily target bactericidal effects or the inhibition of bacterial growth. However, prolonged antibiotic treatments elevate the risk of selection of resistant strains due to selective pressure and the rise of mutants (Husain et al. 2015). In contrast, QSI suppresses bacterial virulence rather than viability, reducing the likelihood of inducing resistance to the QS inhibitors (Bhardwaj et al. 2013).

QSI compounds primarily target the following molecular pathways: (1) Interference with the production of signaling molecules (autoinducers), thereby preventing the activation of the QS system; (2) Inhibition or synthesis of enzymes that modulate the concentration of autoinducers; (3) Competitive inhibition of QS receptors; (4) Modulation of gene expression to prevent the QS system (LaSarre and Federle 2013). In line with these targets and mechanisms of action, EOs and their individual components arise as an option to act on the QS disruption.

**FIGURE 2:**
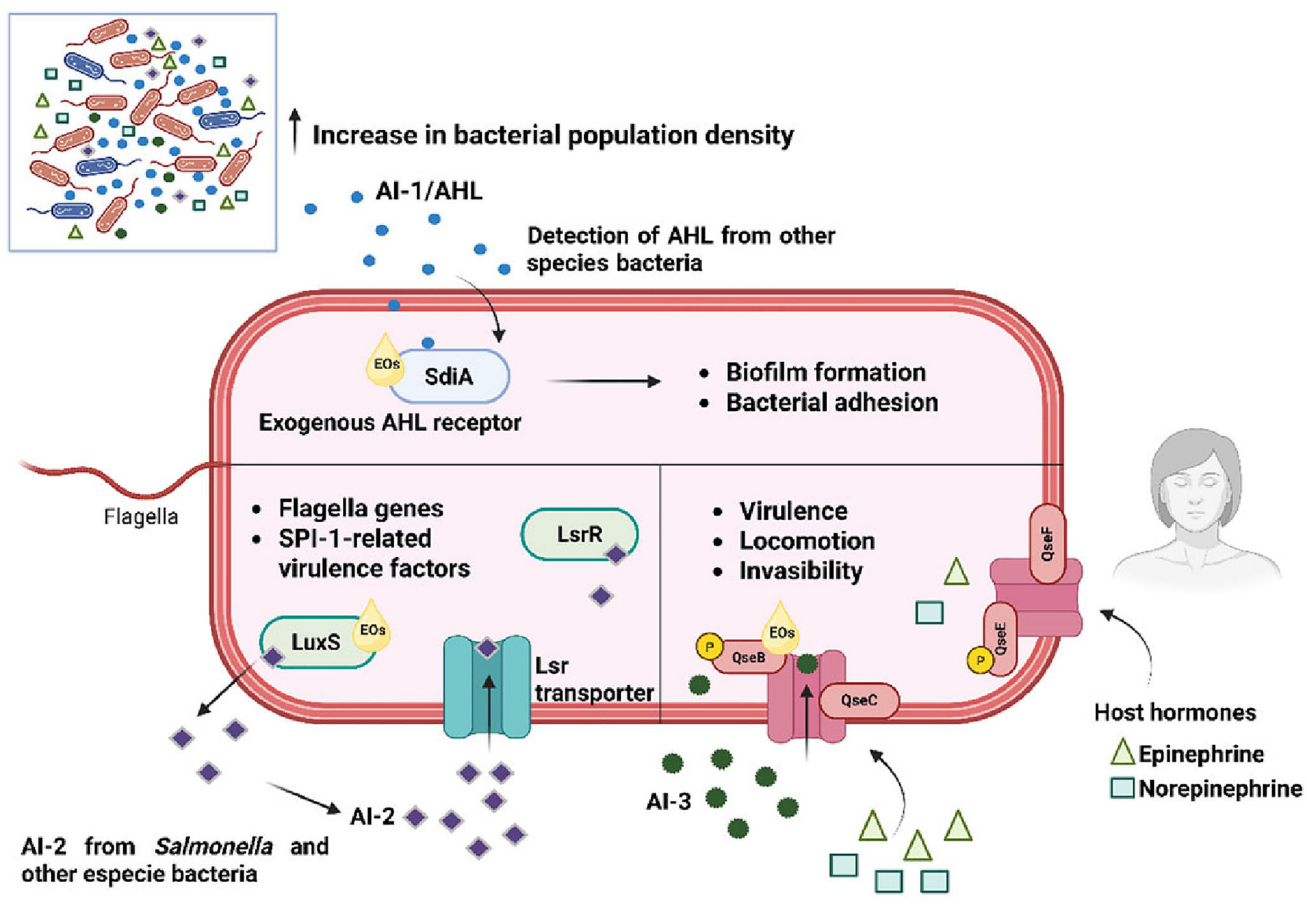
Simplified representation of the main QS systems and their modulation by EOs in *Salmonella* spp. *Salmonella* is capable of detecting various environmental signals, such as autoinducers (AI‐1, AI‐2, and AI‐3) and host hormones (epinephrine and norepinephrine). The SdiA receptor recognizes AHLs produced by other bacteria, regulating biofilm formation and adhesion. AI‐2, synthesized by *Salmonella* and other species, is internalized by the Lsr transporter and modulates the expression of genes related to motility and virulence, with its synthesis depending on the LuxS enzyme. The QseBC system detects AI‐3 and catecholamines, activating genes associated with virulence, motility, and invasion. The QseEF system also responds to Epi and NE and may functionally interact with QseBC. EOs can act as QSIs, downregulating genes such as *luxS*, *pfs*, *sdiA*, and *qseB* (figure created with BioRender).

Currently, various compounds of natural origin are recognized as QSI (Peña‐González et al. 2020). Figure 2 illustrates the mechanism by which EOs interact with QS systems. Hakimi Alni et al. (2020) reported that exposure to the subinhibitory concentration of *Allium sativum* and *Cuminum cyminum* EO negatively regulated the expression of QS system genes (*sdiA* and *luxS*) in *S*. Typhimurium strains isolated from chickens and humans. Similarly, *Artemisia dracunculus* EO sub‐MIC concentrations downregulated the *luxS* and *pfs* genes in an *S*. Typhimurium strain (Pelarti et al. 2021). The Pfs protein encoded by the *pfs* gene is also reported in the literature as a protein necessary for AI‐2 biosynthesis (Beeston and Surette 2002). Ju et al. observed that *S*. Dublin mutants with a knockout of the *luxS* gene had significantly lower AI‐2 production activity than the wild‐type strains (Ju et al. 2018). The enzyme LuxS, encoded by the *luxS* gene, plays an essential role in the synthesis of AI‐2 (Wang et al. 2019). Therefore, the evidence points to subinhibitory concentrations of EOs directly impacting *S*. Typhimurium and *S*. Dublin virulence, as the expression of flagellum‐related genes of the SP‐1 is reduced in the absence of AI‐2 (Choi et al. 2007). Moreover, the downregulation of the *luxS* and *pfs* genes caused by EOs can directly impact biofilm formation. However, evidence suggests that the effect is serotype‐dependent (Mi et al. 2024).

The antimicrobial and antibiofilm effects of *Lippia origanoides* EO were investigated during biofilm formation and QS gene expression in *S*. Enteritidis ATCC 13076 and *S*. Typhimurium ATCC 14028 (Guillín et al. 2021). It was reported that *L. origanoides* EO could inhibit the QS mechanism by negatively regulating *luxS*, *qseB*, and *sdiA* genes (Guillín et al. 2021). QseB is a RR of the QseBC TCS associated with the AI‐3 QS detection. The AI‐3 QS system, which recognizes AI‐3 EPI, NE, is involved in regulating the T3SS and their effectors, is related to the capacity of invading epithelial cells and survival in macrophages (Ji et al. 2017; Moreira et al. 2010). Moreover, the *luxS* gene is related to the AI‐2 type of QS and impairs virulence traits, as already mentioned. Lastly, the *sdiA* gene encodes the SdiA protein, a LuxR homolog that enables the sensing of AHL by other bacteria (Michael et al. 2001). The functions modulated by sdiA in AI‐1 play an essential role in *Salmonella* adhesion, invasion, and biofilm formation, with no significant effect on intracellular survival (Askoura et al. 2021).

EOs can affect bacteria's general pathogenicity and virulence by influencing the expression of genes related to motility (formation of flagella), adhesion (formation of fimbriae), invasion, intracellular survival, and biofilm production (Martínez et al. 2023). These findings suggest that EOs may act as potential QSIs. Exploring and better understanding such compounds is relevant, particularly considering that, according to current evidence, they do not seem to promote mechanisms typically associated with antimicrobial resistance, such as genetic mutations or the selection of resistant subpopulations under selective pressure. Furthermore, no consistent reduction in susceptibility to chemical or physical antimicrobial interventions has been reported in the literature (Leite de Souza 2016). Indeed, studies in the literature even relate synergistic combinations of EOs and conservation treatments such as pulsed UV light (Mutz et al. 2023). Therefore, other methods can adequately achieve microbial inactivation besides its sublethal pathogenicity attenuation effects. It should be highlighted that the combination of treatments should be tested, preferably on food matrices, to assess the antagonist or synergic impacts that can be achieved, besides the matrix effect.

### 3.2 *Salmonella* Biofilm: EOs as Antibiofilm Agents

Microbial contamination poses a serious threat to food safety, especially due to the formation of biofilms (Zhu et al. 2022). These structures protect microbial communities, allowing them to survive in various environments in the food industry and increasing the risk of recurrent cross‐contamination of fresh and processed products (Liu et al. 2024; Zhang et al. 2020). Biofilms are complex structures formed by microorganisms adhered to surfaces and enveloped by an autosecretory matrix of exopolysaccharides (Lin et al. 2017). This structure provides bacteria with a conducive environment to survive and thrive, making them highly resistant to cleaning and disinfection measures, as the exopolysaccharides structure acts as a physical barrier (Yin et al. 2019). The process of biofilm formation can be described in five distinct stages: (1) reversible initial attachment, (2) strong attachment, (3) exopolysaccharides secretion, (4) maturation, and (5) dispersion (Rumbaugh and Sauer 2020; Sharma et al. 2023). The biofilm matrix primarily comprises exopolysaccharides, extracellular DNA (eDNA), and various proteins (Aleksandrowicz et al. 2023; Rumbaugh and Sauer 2020).

Biofilms can develop on abiotic and biotic surfaces, providing a protective environment for bacteria, and the ability of *Salmonella* to form biofilms on different surfaces has been previously reported (Arguello et al. 2012; Lee et al. 2020; Merino et al. 2019; Steenackers et al. 2012). Additionally, the exchange of genetic material increases within the biofilm, and when *Salmonella* develops in these environments, it becomes highly resistant to removal. It can persist even after applying cleaning and intense disinfection methods (Iñiguez‐Moreno et al. 2018). The detaching and resistance features of the biofilm allow for the rapid spread of pathogen contamination throughout food processing plants (Rumbaugh and Sauer 2020). These persistent strains pose a significant risk of cross‐contamination, spreading to other areas and foods, particularly in food processing environments (Liu et al. 2023b). Cross‐contamination refers to the direct or indirect transfer of microorganisms from a contaminated product to an uncontaminated product (Iulietto and Evers 2020). Several studies demonstrate that *Salmonella* strains can contaminate other foods and food preparation areas through cross‐contamination due to personnel handling or contamination of surfaces where multiple foodstuff is processed (da Silva et al. 2023).

Though the constitution may change, in *Salmonella* biofilms, the main constituents of the extracellular matrix include cellulose, curli fimbriae, biofilm‐associated protein (Bap), O‐antigen capsule (O‐Ag‐capsule), lipopolysaccharides, and eDNA (Solano et al. 2002). Among the constituents, curli fimbriae are amyloid nature proteins that play a crucial role in surface adhesion and intercellular interactions (Jonas et al. 2007). They comprise approximately 85% of the biofilm matrix composition of members of the Enterobacteriaceae family, such as *Salmonella* biofilms. Curli fimbriae are fibrillar structures composed of two main subunits, CsgA and CsgB, which significantly contribute to the stability and structuring of the bacterial biofilm (Sleutel et al. 2023). Genes involved in biofilm formation and cell adhesion, such as *adrA*, *bapA*, *csgD*, and the master stress RR *rpoS*, also play a crucial role in the initial attachment or maturation of the biofilm, facilitating cell‐to‐cell interactions (Steenackers et al. 2012).

Flagella, one of the main structures involved in bacterial motility, plays a significant role in the biofilm formation of Gram‐negative bacteria (Ruhal and Kataria 2021). In addition to facilitating the initial contact of the cell with the surface, flagella are also involved in the dispersion of bacterial cells, allowing their colonization in new environments (Benyoussef et al. 2022; Rumbaugh and Sauer 2020; Vilas Boas et al. 2024). Indeed, previous studies involving *S*. Typhimurium *motA* and *fliA* knockout mutants have suggested that motility is important for biofilm development on glass surfaces, as mutants with impaired flagella were unable to form fully developed biofilms (Crawford et al. 2010; Prouty and Gunn 2003).

Several studies have investigated biofilm inhibition formation using chemical methods (Korany et al. 2018). However, many chemical disinfectants can present serious issues for human health and the environment due to their toxic and corrosive effects (Oloketuyi and Khan 2017). Moreover, these strategies can lead to the emergence of highly antibiotic‐resistant strains, representing a significant risk. In this context, the use of natural extracts and phytochemicals has garnered increasing attention as a sustainable and environmentally friendly approach to preventing and eliminating biofilms, particularly in food processing environments. These compounds act by interfering which reduces microbial colonization and persistence. Furthermore, at the applied concentrations, phytochemicals generally present a safe profile for both human health and the environment (Sakarikou et al. 2020). Reports indicate that EOs particularly those rich in terpenoids are among the most promising phytochemical sources, representing a viable and effective alternative to conventional antimicrobials used in the food industry.

Abdullah et al. (2021) assessed the potential of *Amomum subulatum* EO to inhibit biofilm formation in *S*. Typhimurium JSG 1748. The study results showed that the *A. subulatum* EO inhibited biofilm formation at different subinhibitory concentrations (0.03%, 0.06%, 0.12%, 0.25%, and 0.5%) in a dose‐dependent manner. The inhibition of *S*. Typhimurium JSG 1748 biofilm was measured by the crystal violet (CV) stain assay, and inhibitions of 33.67%, 34.14%, 38.66%, 46.65%, and 50.17% were obtained at the respective tested concentrations. It is essential to highlight that most studies in the scientific literature rely on using the CV microtiter assay. This assay evaluates the biofilm‐forming capacity and the efficacy of antibiofilm compounds against bacteria (Ommen et al. 2017). This method is widely used due to its simplicity, reproducibility, and low cost. Typically, these assays are conducted using 96‐well plates, where each well is filled with an appropriate medium and inoculated with the target bacteria. After an incubation period, nonadherent cells are removed by washing, and the formed biofilms are fixed and stained with CV (Kragh et al. 2019; Ommen et al. 2017). The biofilm formed is then quantified by solubilizing the dye in ethanol and/or acetone, followed by an absorbance reading in a spectrophotometer. This protocol allows for an accurate assessment of biofilm density and the efficacy of the tested antibiofilm agents (Kragh et al. 2019; Wilson et al. 2017). Furthermore, in the study mentioned above, the authors reported that compounds tested using a *Chromobacterium violaceum* assay were able to inhibit biofilm formation through QS inhibition (Abdullah et al. 2021). In this assay, violacein production indicates the QS mechanism involved in pigment production. Inhibition of violacein without compromising bacterial growth suggests the presence of a QSI. However, the results of this assay cannot be generalized to other bacteria nor considered definitive proof of QSI.

The use of *C. violaceum* as a reporter bacterium in QS assays is widely embraced due to its distinctive characteristics and practicality. The bacterium's capacity to synthesize the violet pigment violacein in response to QS activation renders it highly valuable (Szabó et al. 2010). This pigment is regulated by the autoinducer molecule (C6HSL), and its distinct coloration enables rapid and precise visual identification of QS activation (McClean et al. 1997). However, it is important to note, as described by Defoirdt et al. (2013), that reporter strains have limitations in assessing anti‐QS effects since the phenotypes evaluated in these assays may be influenced or dependent on other metabolic activities. Consequently, these strains may yield false positive results at a later stage. Additionally, it is essential to exercise caution when extrapolating the findings obtained from this model, considering that *C. violaceum* differs from other bacteria and its specific regulation of QS. Nonetheless, assays with *C. violaceum* are a valuable tool for screening QSI compounds; however, caution should be taken as complementary analysis is necessary to affirm the QSI character (Dimitrova et al. 2023).

In addition to this model‐based approach for studying QS, experimental investigations have also highlighted the potential of plant‐derived compounds in disrupting biofilm formation (Table 2). A study conducted by Pelarti et al. (2021) investigated the efficacy of *Artemisia dracunculus* EO as an antibiofilm agent using the 0.1% safranin staining technique in 96‐well plates. Results showed that EOs prevented the formation of *S*. Typhimurium biofilm at subinhibitory concentrations of 1/2 MIC (1.25 µL/mL) and 1/4 MIC (0.625 µL/mL). Additionally, it was observed in another study that *A. dracunculus* EO can also disrupt mature biofilms already formed by *S*. Typhimurium (Mohammadi Pelarti et al. 2021). Subinhibitory concentrations of thymol (234 and 156 µg/mL) and carvacrol (117 and 78 µg/mL) reduced the bacterial count on the polypropylene surface by approximately 1–2 log of *Salmonella* spp. (*S*. Typhimurium, *S*. Enteritidis, and *S*. Saintpaul). However, a 1–5 log reduction was observed at the MIC or double the MIC for established biofilms.

**TABLE 2:** Targets of biofilm formation affected by EOs and their main compounds on *Salmonella* spp.

Strains	Source of EO	Surface	Concentration	Summary of results	References
*S*. Typhimurium 14028	*L. origanoides*	Polystyrene		Downregulation of genes involved in curli formation (*csgA*, *csgB*, and *csgD)* and motility (*motB*, *flhD*, and *fliZ*)	Guillín et al. (2021)
*S*. Typhimurium isolates (from chicken meat and humans)	*C. cyminum* *A. sativum*	Polystyrene	*C. cyminum*—1/2 MIC (0.98–1.96 µL/mL) *A. sativum* 1/2 MIC (0.25–0.49 µL/mL)	Downregulation of genes involved in cellulose synthesis (*csgD* and *adrA*)	Hakimi Alni et al. (2020)
*S*. Typhimurium 14028	*Coleus amboinicus*	Polystyrene	1/2 MIC (512 µg/mL)	Downregulation of genes associated with motility (*flhD, fljB*, and *fimD*), curli fimbriae production (*csgD*), and invasion (*hilA*)	Leesombun et al. (2023
***S*** *S*. Typhimurium JSG 1748	*A. subulatum*	Polystyrene	0.03%, 0.06%, 0.12%, 0.25%, and 0.5%	Inhibited of biofilm formation in dose‐dependent (33.67%, 34.14%, 38.66%, 46.65%, and 50.17%)	Abdullah et al. (2021)
*S*. Typhimurium 14028	*A. dracunculus*		1/2 MIC (1.25 µL/mL) and 1/4 MIC (0.625 µL/mL)	Inhibited the formation of biofilms and disrupted preformed biofilms	Pelarti et al. (2021)
*S*. Typhimurium 14028 ***S*** *S*. Typhimurium 906‐01 *S*. Enteritidis 502‐00 *S*. Saintpaul 510‐02	Thymol Carvacrol	Polystyrene	Thymol—234 and 156 µg/mL Carvacrol—117 and 78 µg/mL	During biofilm formation, reduced by approximately 1 and 2 log of *Salmonella* spp.	Amaral et al. (2015)
*S*. Derby	Clove EO Oregano EO	Polystyrene	Clove EO—1/2 MIC (0.4 mg/mL and 1/4 MIC (0.2 mg/mL); Oregano EO—1/2 MIC (0.1 mg/mL) 1/4 MIC (0.05 mg/mL)	Inhibition of biofilm formation: Clove EO—1/2 MIC: 92.45 ± 0.31% and 1/4 MIC: 92.28 ± 0.13% Oregano EO—1/2 MIC: 91.15 ± 0.94% and 1/4 MIC: 89.63 ± 1.51%	Liu et al. (2023b)
*S*. Typhimurium 14028	*L. cubeba*	Polystyrene	1/2 MIC (0.4 mg/mL) MBC (0.8 mg/mL)	Biofilm reduction: 6.5% (1/2 MIC), 64.2% (MIC), 84.1% (MBC)	Wang et al. (2025a)
*S*. Typhimurium	*T. vulgaris L*	Polystyrene	1/2 MIC (10 µL/mL) MBC (100 µL/mL)	Inhibition of biofilm formation: 1/2 MIC—30.03 ± 3.52% MBC—89.62 ± 0.32%	Sateriale et al. (2023)
*S*. Typhimurium 14028 *S*. Typhimurium IOC 751/22 ***S*** *S*. Typhimurium IOC 923/15 *S*. Typhimurium IOC 502/22	*O. vulgare* Carvacrol	Polystyrene	*O. vulgare*—1/4 MIC (0.37 mg/mL) 1/2 MIC (0.75 mg/mL) MIC (1.5 mg/mL) Carvacrol—1/4 MIC (0.68–1.37 mg/mL) 1/2 MIC (0.34–0.68 mg/mL) MIC (0.68–1.37 mg/mL)	Inhibition of biofilm formation: *O. vulgare* 1/4 MIC: 64.45 ± 3.60% to 86.19 ± 7.50% 1/2 MIC: 75.23 ± 2.49% to 87.40 ± 2.53% MIC: 82.88 ± 4.48% to 92.88 ± 2.98%) Carvacrol	Ramos et al. (2025)
				1/4 MIC: 44.12 ± 5.35% to 89.64 ± 4.05% 1/2 MIC: 79.53 ± 6.28% to 85.13% ± 1.56% MIC: 84.29 ± 3.55% to 87.80 ± 1.96%)	
*S*. Enteritidis S64	*M. piperita* *C. citratus*	Stainless steel coupons	*C. citratus* MIC (7.80 µL/mL) *M. piperita* MIC MIC (7.80 µL/mL)	Reduction of initially adhered cells: 4.20 and 4.03 log CFU/cm^2^ reductions after 10 min; no detectable cells after 20–40 min	Valeriano et al. (2012)
*S. enterica cocktail (S*. Copenhagen PT99 *S*. Enteritidis CRIFS 1016 *S*. Heidelberg 271 *S*. Kentucky 64701 *S*. Typhimurium 02:8423)		Stainless steel Plastic bottle	MIC (500 µg/mL) 2 × MIC (1000 µg/mL) 4 × MIC (2000 µg/mL)	Max reduction: 1.79 log (steel) and 1.64 log (plastic) at 4 × MIC/30 min; Min reduction: 0.26 log (steel) and 0.58 at 1 × MIC/10 min	Olaimat et al. (2024)

Abbreviations: Max, maximum; MBC, minimum bactericidal concentration; MIC, minimal inhibitory concentration; Min, minimum.

It was observed that clove and oregano EO significantly inhibited the biofilm formation of *S*. Derby at 1/2 and 1/4 MIC (0.2 mg/mL and 0.05 mg/mL) concentrations, as assessed by the CV assay. At the 1/2 MIC concentration, the clove and oregano EO effectively inhibited biofilm formation, with 92.45% and 91.15% inhibition rates, respectively. At the 1/4 MIC concentration, the biofilm formation inhibition rates by clove and oregano EO still reached high values, 92.28%, and 89.63%, respectively (Liu et al. 2023b). Scanning electron microscopy (SEM) and confocal laser scanning microscopy (CLSM) images showcased significant inhibitory effects of clove and oregano EO at 1/2 MIC and 1/4 MIC on the bacterial biofilm and its structure. Furthermore, transcriptomic analyses revealed a downregulation of genes involved in the degradation of long‐chain fatty acids, which are fundamental for the bacterium's energy metabolism. The EOs suppressed the expression of critical genes in this process, affecting the synthesis of intermediates in the tricarboxylic acid (TCA) cycle and oxidative phosphorylation, significantly reducing ATP production and enzymatic activity (Liu et al. 2023b). These findings indicate that EOs can suppress the energy metabolism of *S*. Derby's biofilm, contributing to the inhibition of bacterial biofilm formation.

Assessing the antibiofilm activity of *L. origanoides* EO in strains of *S*. Typhimurium ATCC 14028 and *S*. Enteritidis ATCC 13076 revealed an inhibition of approximately 60% in biofilm formation in both strains, as determined by CV assay (Guillín et al. 2021).

According to the authors, this inhibitory effect may be related to the EO's ability to reduce bacterial motility and disrupt the production of surface structures such as adhesins, curli proteins, and flagella (Guillin et al. 2021; Toyofuku et al. 2016). This hypothesis is supported by transcriptional assays, which showed that *L. origanoides* EO downregulated genes involved in the of curli fibers (*csgA*, *csgB*, *csgD*) and those associated with motility (*motB*, *flhD*, *fliZ*; Guillin et al. 2021). Similarly, *Coleus amboinicus* EO has also demonstrated the ability to suppress motility‐related genes, including *flhD*, *fljB* (involved in flagellar biosynthesis), and *fimD* (Leesombun et al. 2023).

In addition to effects on motility and adhesion structures, other EOs such as those of *Cuminum cyminum* and *Allium sativum* have been shown to downregulate genes associated with cellulose synthesis when used at 1/2 MIC concentrations against *S*. Typhimurium isolates, further supporting the role of EOs in targeting biofilm formation at the genetic level (Hakimi Alni et al. 2020).

In this context, the antibiofilm effects of EOs demonstrate outstanding potential for application in the food industry, especially given the risk of microbial contamination that can occur at all stages of production, transport, and marketing (Yuan et al. 2021). *Salmonella* strains are capable of adhering to various material surfaces, such as stainless steel, plastic, rubber, and glass (Pavone et al. 2025; Wang et al. 2025a). Given the impact of biofilms on food safety, several strategies have been developed to prevent or inhibit their formation. These methods include physical sterilization (such as UVC radiation, heat, and ultrasound) and chemical treatments (disinfectants and sanitizers; Gao et al. 2023). However, the improper use of chemical treatments may result in physiological changes in bacterial cells, which could promote biofilm production (Yuan et al. 2021). Furthermore, these approaches can induce stress responses in bacteria, making them more resistant to antimicrobial treatments.

For that reason, to the prevention biofilm formation through alternative methods, such as the application of EOs is of great importance. For example, thyme EO demonstrated strong antimicrobial effects, significantly inhibiting biofilm formation of *S*. Typhimurium and *B. cereus* in a dose‐dependent manner for concentrations between 10 and 100 µg/mL in 96‐well polyethylene plates (Sateriale et al. 2023). Similarly, for 96‐well polyethylene plates, Ramos et al. (2025) observed antiadhesive activity of *O. vulgare* EO and carvacrol at different concentrations (1/4 MIC, 1/2 MIC, and MIC) in *S*. Typhimurium, finding a significant inhibition of 86.19% at the 1/4 MIC (0.37 mg/mL).

Additionally, the ability to destroy already established biofilms was also evaluated after treatment with the EOs. A sanitizing formulation based on *Mentha piperita* and *Cymbopogon citratus* EOs showed efficacy in controlling biofilms of S. Enteritidis, with significant logarithmic reductions of 4.20 and 4.03 in biofilm cells, respectively, after 10 min of treatment on stainless steel coupons. After 20 and 40 min of exposure, both EOs completely eliminated the biofilm, with cell counts below the detection limit (< 0.03 CFU/cm^2^; Valeriano et al. 2012). Olaimat et al. (2024) reported a reduction of *S. enterica* cells in preformed biofilms. The Aleppo pine (*Pinus halepensis*) EO reduced up to 1.79 ± 0.12 log CFU of *S. enterica* cells in biofilms on stainless steel surfaces after 30 min of treatment. After 20 min, the reduction was 1.35 ± 0.06 log CFU, and after 10 min, 1.04 ± 0.10 log CFU/coupon. On plastic surfaces, the reductions observed were 1.64 ± 0.07 log CFU after 30 min, 1.31 ± 0.09 log CFU after 20 min, and 1.08 ± 0.05 log CFU after 10 min. These results are significant because the ability of pathogens to adhere and form biofilms on stainless steel or plastic surfaces has become an increasing concern globally, especially in the food industry.

Although evidence shows the ability of EOs to inhibit biofilm formation and act on adhered cells, it is essential to highlight that their application on different surfaces may present some limitations, such as low solubility, volatility, and poor adhesion (McClements et al. 2021). Therefore, there is a search for new, effective, and innovative techniques. Nanoemulsification emerges as a promising alternative to improve the dispersion, stability, and bioavailability of EOs. In their study, Liu et al. (2022) observed that a nanoemulsion of garlic EO showed greater stability and was significantly more effective in inhibiting the growth of *S. aureus* compared to pure garlic EO.

Additionally, subinhibitory concentrations (1/4 and 1/2 MIC) of *O. vulgare* and carvacrol nanoemulsions demonstrated superior or equivalent antiadhesive activity compared to pure EO against *S*. Typhimurium strains on polystyrene microplates (Ramos et al. 2025). The nanoemulsion developed from *trans*‐cinnamaldehyde (from cinnamon bark) also reduced biofilm formation of *S*. Enteritidis on polystyrene and stainless steel plates. Moreover, effects on the inactivation of mature biofilms were observed, with the nanoemulsion showing higher inactivation efficacy compared to the oily form in various dose and time combinations (Shah et al. 2025).

These enhanced effects are likely attributed to the more uniform dispersion of the compound in nanoemulsion form. Additionally, nanoemulsions have a higher surface area‐to‐volume ratio than pure EO, facilitating more effective interactions with target sites and increasing biological activity (da Silva et al. 2023; McClements et al. 2021). Moreover, applying nanoemulsions as coatings has shown promise in food preservation, contributing to the shelf‐life extension of fresh and meat products (da Silva et al. 2023; Hasan et al. 2020). These results are promising, as they indicate the potential for enhanced EOs effects, making them more effective for application on surfaces and in controlling or reducing biofilm formation on typical food processing surfaces such as plastic and stainless steel.

**FIGURE 3:**
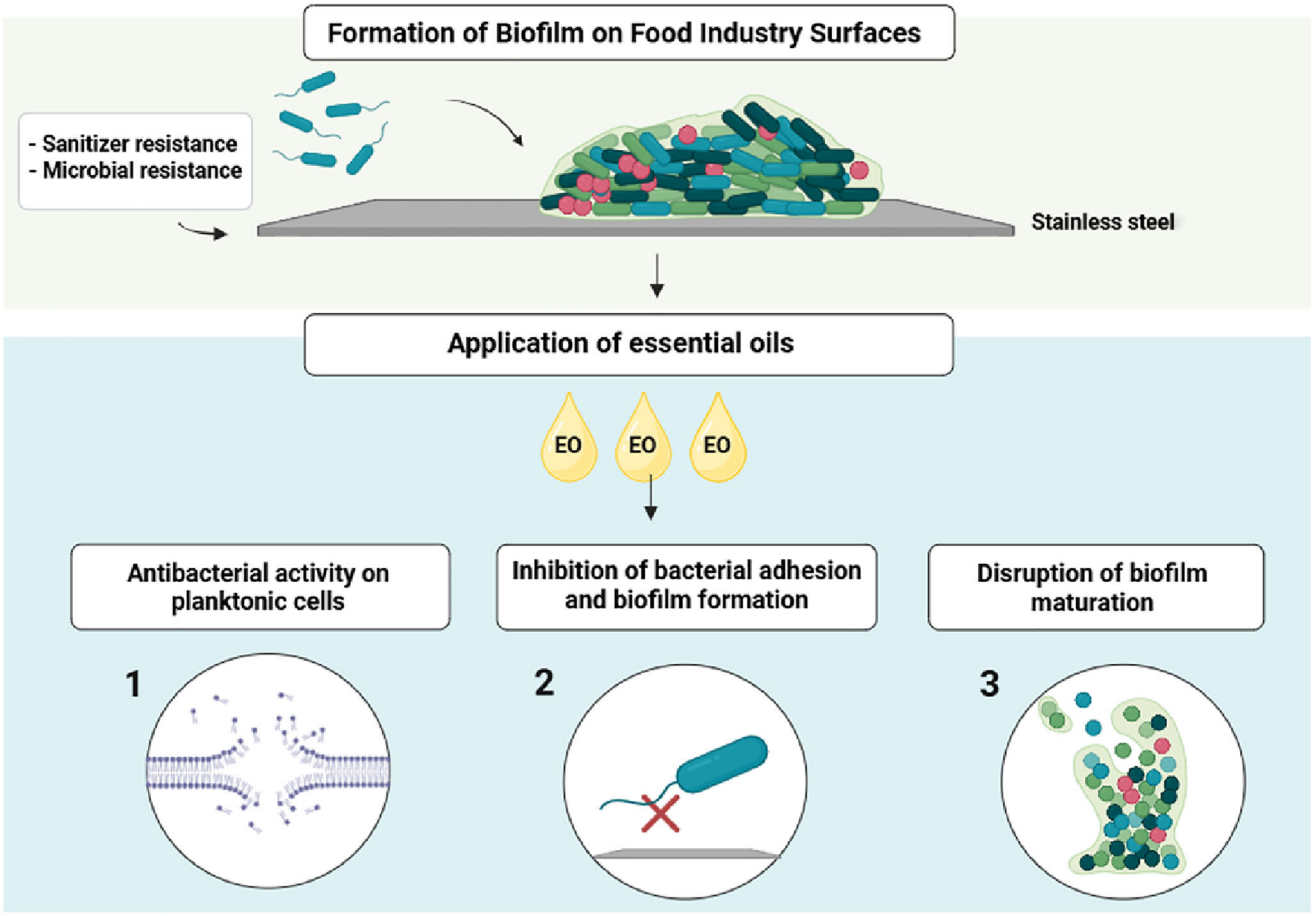
Biofilm formation on food industry surfaces and the application EOs to mitigate the effects of this formation. The image illustrates the process of bacterial adhesion to stainless steel surfaces, leading to biofilm formation, which results in sanitizer resistance and Microbial resistance. The application of EOs acts in different ways: (1) antibacterial activity on planktonic cells, (2) inhibition of bacterial adhesion to the surface, and (3) disruption of biofilm maturation (figure created with BioRender).

Therefore, these results suggest that using EOs could be an effective alternative against the biofilm problem in the food industry, applicable to various surfaces. In terms of practical applications, subinhibitory concentrations of EOs or nanoemulsions could be integrated into existing food safety protocols, combined with already implemented antimicrobial methods in lower doses, as part of pathogen hurdle strategies control (Figure 3). However, despite the promising results, it is essential to highlight that variations in the concentrations and antimicrobial efficacy of EOs among studies indicate the need for further standardization in the methodologies used. Thus, practical applications should be preceded by an adequate methodology for the specific case scenario.

### 3.3 Efflux Pump‐Mediated Resistance in *Salmonella*: EOs as Efflux Pump Inhibitors

Gram‐negative bacteria have a cell envelope structure with two membranes: the cytoplasmic membrane, made up of a phospholipid bilayer, and the outer membrane, composed of phospholipids in the inner leaflet and lipopolysaccharides in the outer leaflet. Between them lies the periplasmic space, containing peptidoglycan and periplasmic proteins (Schwechheimer and Kuehn 2015). Embedded within these membranes are protein channels responsible for transporting substances, either for uptake or efflux (Nishino et al. 2009). Efflux pumps actively transport a variety of antibiotics out of the cell. Some pumps are specific for a single substrate, while others can transport a broad range of structurally distinct substrates (Blair et al. 2015). It has been shown that the overproduction of the AcrAB pump in *E. coli* and *S*. Typhimurium leads to resistance against dyes, detergents, disinfectants, and bile salts (Giraud et al. 2000). AcrAB is an essential transporter, while the porin OmpF is a primary influx channel that selectively allows the entry of antibiotics into the cell (Alenazy 2022).

Most bacteria possess multiple genes encoding these proteins. These categories include the ABC (ATP‐binding cassette) family, multidrug and toxic compound efflux (MATE) transporters, the small multidrug resistance (SMR) family, members of a larger drug/metabolite transporter superfamily, resistance‐nodulation‐cell division (RND) proteins, and the major facilitator superfamily (MFS; Alenazy 2022; Nishino et al. 2009). In *Salmonella* strains, the efflux system consists of 10 pumps belonging to four families (ABC, MATE, MFS, and RND; Alenazy 2022). Efflux pumps of the RND family act as polyspecific transporters and are directly responsible for multidrug resistance in *Salmonella*. AcrAB‐TolC, belonging to the RND efflux system, is the most abundant efflux pump in *S*. Typhimurium (Colclough et al. 2020). AcrB forms homotrimers in the inner membrane, constituting a tripartite complex with the periplasmic adapter protein AcrA and the outer membrane channel TolC (Blair et al. 2015). AcrB can expel various compounds, including tetracyclines, hydrophobic fluoroquinolones (such as norfloxacin and ciprofloxacin), and bile acids (Blanco et al. 2016).

Efflux pump inhibitors (EPIs) have emerged as a promising strategy to enhance the action of antibiotics against multidrug‐resistant (MDR) strains, preventing the extrusion of substances and favoring their intracellular accumulation (Reza et al. 2019). In addition, EPIs can modulate regulatory signals, inhibiting these pumps' expression and functional assembly of these pumps (Seukep et al. 2022). Other mechanisms include collapsing the energy required for efflux and directly blocking the transport of substances (Anndressa et al. 2021; Pagès and Amaral 2009). Thus, EPIs represent a promising approach to combat antimicrobial resistance and the spread of resistant bacteria.

**FIGURE 4:**
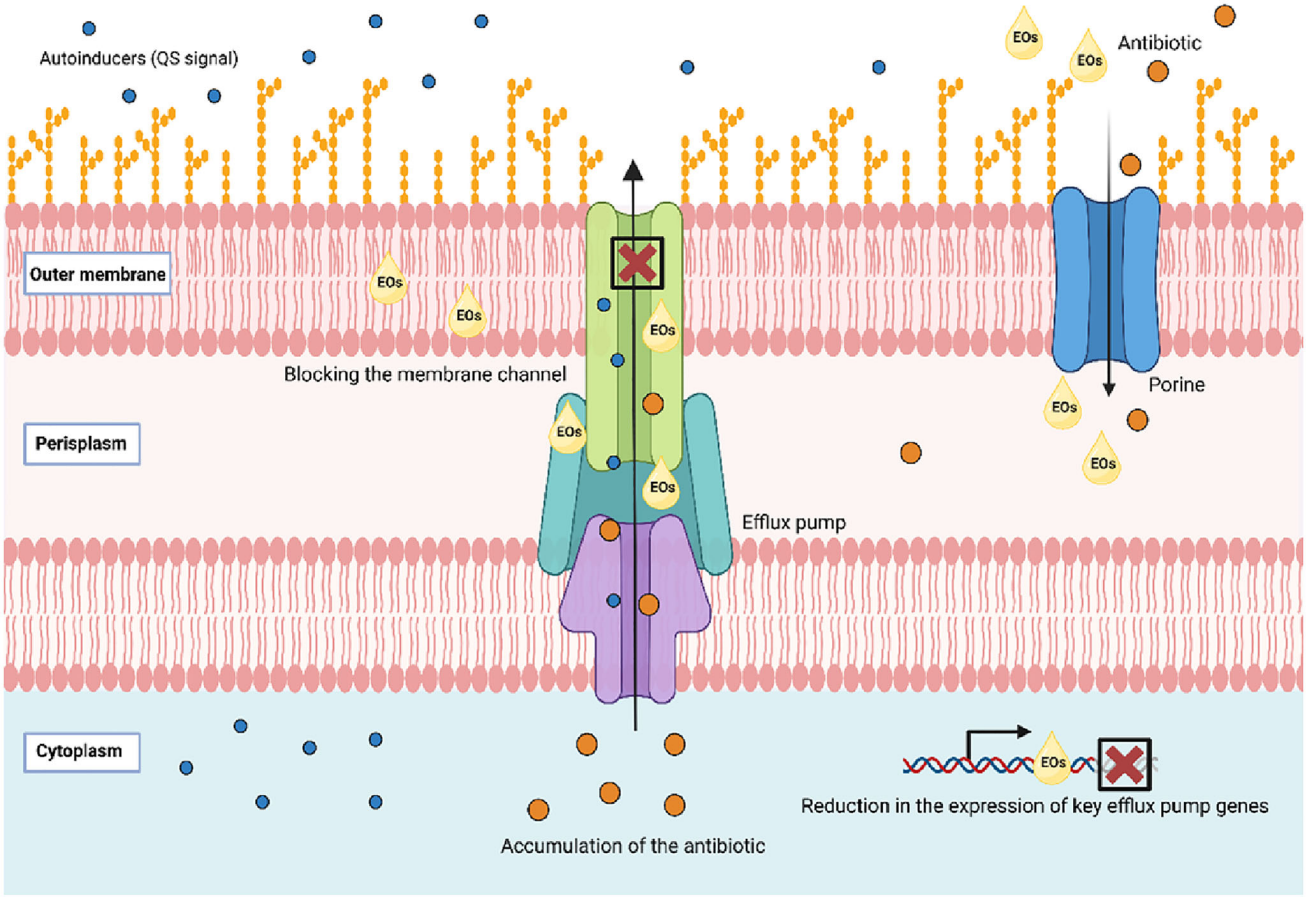
EOS can act by blocking efflux pump, thereby preventing the extrusion of antibiotics and promoting their accumulation within the bacterial cell. These compounds can also interfere with the regulation of genes expression efflux pump, reducing their activity. Furthermore, EOs can inhibit biofilm formation by hindering the transport of QS autoinducers to the extracellular.

EOs have gained attention for their antimicrobial properties, including the inhibition of efflux pumps, preventing the expulsion of antibiotics (Anndressa et al. 2021). The efflux activity of *Salmonella* exposed to *Mentha arvensis* (MAEO; 0.625 µL/mL) was assessed by flow cytometry using the ethidium bromide (EB) efflux assay (de Sousa Guedes and de Souza 2018). This dye, which is usually expelled by active transport, accumulates inside cells with compromised efflux pumps, binding to DNA (Díaz et al. 2010; Kim et al. 2009). Exposure to MAEO compromised the activity of these pumps, increasing the percentage of EB‐labeled cells in pineapple juice from 10.1% to 71.8% compared to the control (de Sousa Guedes and de Souza 2018). Similar results were observed by Lira et al. (2020), who investigated the potential of EOs from OVEO and *R. officinalis* L. (ROEO) in inhibiting the efflux pump activity of *S*. Enteritidis 86. After 15 min of exposure to OVEO (2.5 µL/mL) and ROEO (40 µL/mL), efflux activity was reduced by 78% and 84%, respectively. The inhibition of these efflux pumps using EOs can increase the intracellular concentration of antibiotics or other antimicrobial agents, enhancing their effectiveness against *Salmonella* strains (Figure 4).

In a study conducted by El‐Demerdash et al. (2024), it was demonstrated that components of EOs, such as cumin and cinnamon, inhibit the AcrAB and AcrD pumps of MDR *Salmonella*. Treatment with these EOs significantly reduced the expression of key efflux pump genes (*robA*, *acrB*, *mdtB*, *acrF*, *acrD*, *soxS*, *mdsB*, *marA*). The inhibition of these genes may prevent the formation of efflux pumps, suggesting that these natural compounds can increase the intracellular concentration of antibiotics and, consequently, enhance their effectiveness.

On the other hand, studies with *E. coli* O157:H7 adapted to subinhibitory concentrations of thymol and carvacrol showed an increase in the expression of efflux genes (*marA* and *acrB*), although the functional activity of these pumps was reduced (Yuan and Yuk 2019). This suggests that while some EOs may act by directly inhibiting the expression of efflux genes, others may induce their expression as a stress response but compromise their functionality by interacting with the transporter proteins or depleting cellular energy.

Additionally, evidence suggests that EPIs can interfere with communication systems mediated by QS (Dawan et al. 2022). The involvement of efflux pumps in QS was first observed in *Pseudomonas aeruginosa*, where the MexAB‐OprM pump transports extracellular signals (Pearson et al. 1999; Subhadra et al. 2018). QS regulates virulence factors and biofilm formation (Rutherford and Bassler 2012), and its inhibition can compromise bacterial persistence. Biofilm formation capacity was reduced after the deletion of efflux pump genes such as *acrD*, *acrEF*, *emrAB*, *macAB*, *mdfA*, *mdsABC*, *mdtBC*, *mdtK*, and *tolC* in *S. enterica* (Alenazy 2022; Baugh et al. 2012).

Miladi et al. (2017) evaluated the antibacterial susceptibility and biofilm eradication of carvacrol, thymol, and eugenol, alone or in combination with nalidixic acid, against 12 strains of *S*. Typhimurium. All tested compounds demonstrated a synergistic effect with nalidixic acid in biofilm eradication, significantly reducing the MBEC50 of the acid (13.9–188 µg/mL). Thymol was the most effective, followed by eugenol and carvacrol. Additionally, the three compounds significantly inhibited the efflux pumps, with concentrations for 50% inhibition of efflux of 190 µg/mL for carvacrol, 173 µg/mL for thymol, and 230 µg/mL for eugenol. These results indicate that phenolic compounds increase the permeability of the bacterial membrane and induce greater susceptibility to antibiotics by inhibiting efflux pumps, favoring biofilm eradication.

Despite the promising findings on the effects of EOs on efflux systems, there is a significant gap in the scientific literature regarding using EOs as EPIs in *Salmonella*. Few studies have detailed the specific impacts of subinhibitory concentrations of EOs and their chemical components on the efflux pumps of this bacterium. This highlights the need for more comprehensive and detailed research on the potential of EOs to inhibit these bacterial resistance mechanisms.

### 3.4 *Salmonella* Antimicrobial Resistance Versus EOs Effectiveness

Antibiotic resistance is defined as the ability of microorganisms to survive and multiply even in the presence of antimicrobial agents that would normally inhibit their growth (Pulingam et al. 2022). This occurs through mechanisms such as alterations in membrane permeability, enzymatic inactivation, modification of target sites, and restructuring of the cell membrane (Hu et al. 2020). Additionally, resistance genes are often acquired horizontally through transformation, transduction, and bacterial conjugation (Pulingam et al. 2022). The excessive use of antibiotics in human medicine, veterinary medicine, and animal production is a key factor in spreading resistance genes (Bhandari et al. 2023)

In the case of *Salmonella*, an increase in resistance to first‐line antibiotics such as ciprofloxacin and ceftriaxone has been observed, compromising the efficacy of these drugs in clinical settings (Gangathraprabhu et al. 2020). Although alternatives such as ampicillin, chloramphenicol, and trimethoprim‐sulfamethoxazole are used, the emergence of MDR strains remains a global concern (Marchello et al. 2020).

To overcome these challenges, combining EOs with conventional antibiotics has been proposed as a promising strategy to enhance antimicrobial efficacy. These combinations often result in synergistic effects, allowing for reduced antibiotic concentrations without compromising, and even enhancing, antimicrobial activity (Langeveld et al. 2014; Zahli et al. 2023).

EOs contain bioactive phytochemicals, such as terpenes, phenols, tannins, flavonoids, and alkaloids, with various mechanisms of action (Table 1). These compounds can destabilize bacterial membranes, interfere with protein synthesis, and inhibit efflux pumps, among other effects (Dias et al. 2022). Although some of these compounds show modest antibacterial activity when used alone, their combination with antibiotics can increase their therapeutic effects synergistically (Seukep et al. 2020; Shriram et al. 2018).

One of the relevant mechanisms is inhibiting bacterial efflux pumps, which are responsible for expelling antibiotics from the cell, reducing their intracellular concentration (Section 3.3). For example, Miladi et al. (2016) revealed that adding thymol or carvacrol to tetracycline (TET) reduced the MIC of this antibiotic against *Salmonella* strains. At 250 and 500 µg/mL concentrations, these compounds inhibited efflux activity, as confirmed by EB assays, leading to intracellular accumulation of TET.

Table 3 summarizes the synergistic effects between EOs and antibiotics against *Salmonella* spp. A study evaluated the resistance of 36 *S. enterica* strains to antibiotics and various EOs (*Thymus vulgaris*, *Eugenia caryophyllata*, and *Corydothymus capitatus*; Lauteri et al. 2022). The combination of tetracycline with the *C. capitatus*, *E. caryophyllata*, and *T. vulgaris* EO significantly reduced the MIC values of tetracycline from 256 to 4 mg/mL, suggesting a restoration of susceptibility to the antibiotic (Lauteri et al. 2022). These findings indicate that EOs represent a promising alternative in combined therapy against MDR pathogens.

**TABLE 3:** Antimicrobial interactions between EOs and antibiotics against *Salmonella* spp.

*Salmonella* strain	EO or component	Antibiotic	Results	References
*S*. Typhimurium *S*. Enteritidis *S*. Rissen *S*. Typhy *S*. Derby	*T. vulgaris* *Eugenia caryophyllata* *Corydothymus capitatus*	Tetracycline	Mostly additive effect between tetracycline and EOs of *C. capitatus*, *E. caryophyllata*, and *T. vulgaris*. Synergistic interactions observed in up to 9.6% of strains. Indifferent effect in some cases; antagonism in only one strain.	Lauteri et al. (2022)
*S*. Kentucky *S*. Typhimurium *S*. Chester *S*. Schwarzengrund *S*. Enteritidis ATCC 13076	*T. capitatus* *S. aromaticum*	Ampicillin Gentamicin	Total synergy observed in 94% (34/36) of combinations between *T. capitatus* and *S. aromaticum* EOs with ampicillin and gentamicin; partial synergy in one case (*S. aromaticum* + gentamicin vs. *S*. Typhimurium).	Zahli et al. (2023)
*S*. Kentucky *S*. Chester *S*. Typhimurium *S*. Schwarzengrund	*O. elongatum* *O. compactum*	Ampicillin Gentamicin	*O. elongatum* + gentamicin was synergistic in 88% of strains; *O. compactum* + gentamicin showed lower activity (five additive, four synergistic); For ampicillin combinations, 78% (14/18) were synergistic, with *O. elongatum* + ampicillin showing the strongest effect.	Zahli et al. (2024)
*Salmonella* spp.	*Cladanthus arabicus Bubonium imbricatum*	Amoxicillin Neomycin	Total synergy of *C. arabicus* and *B. imbricatum* EOs with amoxicillin; No synergistic interaction with EOs and neomycin.	Aghraz et al. (2018)
*S*. abony ATCC 6017	*Calamintha sylvatica* *C. vardarensis* *C. nepeta*	Gentamicin Ciprofloxacin	Total synergy was observed in the combinations between EOs and gentamicin or ciprofloxacin, except for *C. glandulosa*, which showed an additive effect with the antibiotics.	Milenkovic et al. (2018)
*S*. Typhimirium SGI1 (tetA)	Carvacrol Thymol Eugenol Cinnamaldehyde Allyl isothiocyanate	Tetracycline Ampicillin Penicillin G Erythromycin Bacitracin Novobiocin	Thymol was synergistic with all antibiotics except erythromycin; Carvacrol was synergistic with all antibiotics except with ampicillin and erythromycin. Eugenol was synergistic with tetracycline and novobiocin. Cinnamaldehyde showed synergy with most antibiotics, except penicillin. AIT showed no interaction with tetracycline, penicillin, or novobiocin.	Palaniappan and Holley (2010))

Additional synergistic effects were observed when the of EOs *T. capitatus* and *Syzygium aromaticum* were combined with gentamicin and ampicillin against nine *Salmonella* strains. Eight of these strains were MDR, isolated from food (*S. Kentucky*, *S*. Typhimurium, *S. Chester*, *S*. Schwarzengrun), and one was a reference strain (*S*. Enteritidis ATCC 13076; Zahli et al. 2023). The checkerboard test was used for this analysis, a widely employed in vitro technique for evaluating synergy or antagonism between substances. This method arranges the concentrations of one substance horizontally and those of the other vertically on a microplate. The concentrations tested are based on the MIC of the substances, ranging from values below to twice the expected MIC (Langeveld et al. 2014). Among the 36 combinations tested of *T. capitata* and *S. aromaticum* EO with ampicillin and gentamicin, 34 (94%) showed complete synergy. One combination showed partial synergy (*S. aromaticum* EO with gentamicin against *S*. Typhimurium), and another showed no interaction (*S. aromaticum* EO with gentamicin against *S. Kentucky*). The combination of *T. capitata* EO with ampicillin had the best antibacterial effects, with fractional inhibitory concentration indices (FICi) ranging from 0.018 to 0.5. The FICi index allows for assessing the type of interaction between substances, indicating synergy (FICi ≤ 0.5), additivity (0.5 > FICi ≤ 1), indifference (1 > FICi ≤ 4), or antagonism (FICi > 4; Langeveld et al. 2014; Mantzana et al. 2023). Additionally, some authors also consider the term “partial synergy,” which refers to an FICi between 0.5 and 0.75 (Zahli et al. 2023).

Complete synergistic effects were observed against all *Salmonella* strains, including the reference strain, when *T. capitata* EO was combined with ampicillin or gentamicin. For *S. aromaticum* EO, total synergy (FICi from 0.12 to 0.5) and partial synergy (FICi = 0.51) with the antibiotics were observed, with FICi values between 0.125 and 0.5 for ampicillin and between 0.14 and 0.51 for gentamicin. Among the 18 combinations between *S. aromaticum* EO and the antibiotics, one showed no interaction with gentamicin against *S. Kentucky* (Zahli et al. 2023). In another study Zahli et al. (2024) investigated the interactions of the combination of *O. elongatum* and *O. compactum* with ampicillin and gentamicin against *Salmonella* strains. The combination of *O. elongatum* + gentamicin stood out, showing synergy in 88% of the strains analyzed.

EOs may compromise membrane integrity, facilitating the access of antibiotics to intracellular targets. For example, the enhanced uptake of gentamicin, an antibiotic that inhibits mRNA translation by binding to the 16S rRNA of the 30S ribosomal subunit, may be facilitated by increased permeability induced by EOs (Yoshizawa et al. 1998). Furthermore, EOs can act on the same action site as antibiotics, as is the case with amoxicillin, a beta‐lactam that inhibits bacterial cell membrane synthesis, similarly to EOs. Thus, the combination of these substances can potentiate antimicrobial effects. Indeed, Aghraz et al. (2018) observed total synergistic interaction between the EOs of *Cladanthus arabicus* and *Bubonium imbricatum* with amoxicillin; the combination of amoxicillin with *B. imbricatum* EO increased antimicrobial activity against *Salmonella* spp. fourfold. Similarly, (Palaniappan and Holley 2010) found that carvacrol and thymol, at subinhibitory concentrations, enhanced the effectiveness of several antibiotics, including tetracycline, erythromycin, and bacitracin, against *S*. Typhimurium SGI1.

These findings reinforce the potential of combining EOs and antibiotics in vitro. The synergy generated by multiple mechanisms, including efflux pump inhibition, membrane disruption, and metabolic interference, can restore or significantly enhance the effectiveness of antibiotics against *Salmonella*. This presents promising implications, with great potential for facing the public health burden of MDR pathogens.

However, despite the potential benefits, in the practical aspects, there are still no studies on viabilization of this combination. Data are still needed, especially regarding the toxicity and volatility of EOs, which may limit their direct use (Fuentes et al. 2021). To overcome these limitations, innovative strategies such as nanoencapsulation, controlled release systems, and targeted delivery might be one way to improve bioavailability and reduce toxicity, and need to be explored (Nishitani Yukuyama et al. 2017; Shi et al. 2024). Furthermore, in vivo studies and clinical trials are urgently needed to validate the promising results obtained in in vitro studies (Janotto et al. 2023). The establishment of standardized protocols for EO–antibiotic combination testing, dosage optimization, and evaluation of interactions with human cells are crucial steps for advancing this approach and its application.

## 4 Conclusion and Future Perspectives

EOs are potent antimicrobial agents, showing activity against *Salmonella* even at subinhibitory concentrations. EOs act on various molecular targets, such as cell membranes, efflux pumps, and QS systems, showing efficacy in inhibiting biofilm formation and reducing bacterial virulence, even at subinhibitory concentrations. These properties can be used in combination with antibiotics to enhance their efficacy and reduce bacterial resistance. Although the results are promising, there are still challenges, such as the volatility and sensory impact of EOs, as well as inconsistencies in the data on bacterial resistance after subinhibitory exposure. Combining EOs with additional technologies, such as nanoemulsions, may help overcome these limitations. Future research should investigate the long‐term effects of subinhibitory concentrations on resistance mechanisms and the feasibility of using EOs in combined pathogen control strategies.

## Author Contributions

**Carolina Ramos**: conceptualization, methodology, writing – original draft. **Leticia Tessaro**: conceptualization, methodology. **Bruno Dutra da Silva**: writing – original draft, writing – review and editing. **Marciane Magnani**: writing – review and editing, writing – original draft. **Carlos Adam Conte‐junior**: writing – review and editing, visualization, software, supervision, writing – original draft. **Yhan S. Mutz**: conceptualization, investigation, methodology, writing – review and editing, writing – original draft

## Conflicts of Interest

The authors declare no conflicts of interest.
